# Discovery of novel mutations in the dihydropyrimidine dehydrogenase gene associated with toxicity of fluoropyrimidines and viewpoint on preemptive pharmacogenetic screening in patients

**DOI:** 10.1186/s13167-015-0039-x

**Published:** 2015-09-02

**Authors:** Marzia Del Re, Angela Michelucci, Angelo Di Leo, Maurizio Cantore, Roberto Bordonaro, Paolo Simi, Romano Danesi

**Affiliations:** Division of Pharmacology, Department of Clinical and Experimental Medicine, University of Pisa, 55, Via Roma, 56126 Pisa, Italy; Cytogenetics and Molecular Genetics Unit, University Hospital, Pisa, Italy; Medical Oncology Unit, ASL4, Prato, Italy; Medical Oncology Unit, Azienda Ospedaliera Carlo Poma, Mantova, Italy; Medical Oncology Unit, Azienda Ospedaliera Garibaldi, Catania, Italy

**Keywords:** Prediction, Personalization, Medicine, Toxicity, Colorectal cancer, Fluoropyrimidines, DPD

## Abstract

**Background:**

Dihydropyrimidine dehydrogenase (DPD) is the initial and rate-limiting enzyme of the metabolic pathway of 5-fluorouracil (5-FU) and other fluoropyrimidines to inactive compounds. For this reason, severe, life-threatening toxicities may occur in patients with deficient DPD activity when administered standard doses of 5-FU and its prodrugs.

**Materials and methods:**

We selected three patients with colorectal adenocarcinoma who displayed unexpected severe adverse reactions after treatment with 5-FU and capecitabine. To investigate the possible involvement of deficient variants of the DPD gene (*DPYD*), a denaturing HPLC (dHPLC) approach followed by target exon sequencing of *DPYD* was performed on DNA extracted from peripheral blood.

**Results:**

Three novel non-synonymous mutations of *DPYD*, c.2509-2510insC, c.1801G>C, and c.680G>A, were detected in these subjects. Due to the absence of other deficient variants of *DPYD* and the compatibility of adverse reactions with fluoropyrimidine treatment, the novel variants were associated with a poor-metabolizer phenotype.

**Conclusions:**

Stratification of patients on the basis of their genotype may help prevent toxicity, and the large body of evidence about the pathogenesis of fluoropyrimidine-induced adverse reactions strongly encourages the adoption of best practice recommendations to appropriately address this important clinical issue. This approach is of utmost importance within a preventive, prognostic, and personalized approach to patient care in the oncology setting.

## Overview

Fluoropyrimidines, including 5-fluorouracil (5-FU) and its prodrugs capecitabine and ftorafur/uracil (UFT), are used for the treatment of solid tumors including colorectal, breast, pancreatic, and head and neck cancers [1]. Capecitabine (N^4^-pentyloxycarbonyl-5′-deoxy-5-fluorocytidine) is an orally administered fluoropyrimidine, which undergoes extensive metabolism to 5-FU via a three-step enzymatic process. Capecitabine is first hydrolyzed by carboxylesterase in the liver to 5′-deoxy-5-fluorocytidine (5′-DFCR) and then metabolized to 5′-deoxy-5-fluorouridine (5′-DFUR) by cytidine deaminase. 5′-DFUR is finally converted to 5-FU by thymidine phosphorylase [2]. The initial rate-limiting step of the inactivation of 5-FU to dihydrofluorouracil occurs via dihydropyrimidine dehydrogenase (DPD), a ubiquitous enzyme mostly represented in the liver. Mutations in the DPD gene (*DPYD*) may compromise its activity and cause severe toxicity in patients given standard doses of fluoropyrimidines [3]. A number of genetic variants of *DPYD* associated with the deficient phenotype have been described [4], although the full spectrum of mutations is unknown thus far. The knowledge of the poor-metabolizer status in the individual patient is mandatory to allow safe administration of chemotherapeutic drugs to meet the general principle of preventive, prognostic, and personalized medicine for optimal oncology care. This article reports on three novel mutations of *DPYD* in patients treated with 5-FU or capecitabine and experiencing unexpected life-threatening toxicities and discusses the importance of developing best clinical laboratory practice recommendations for the use of preemptive genetic screening of *DPYD*.

## Methods

Blood samples were obtained from a peripheral vein of three patients with severe toxicities, as described below, and DNA extraction was performed using Qiagen Biorobot® (Qiagen, CA, USA). The full coding region of *DPYD* was amplified by PCR, and the presence of sequence variants was screened by denaturing high-performance liquid chromatography (dHPLC; Transgenomic, CT, USA). dHPLC uses heteroduplex formation between wild-type and mutated DNA strands to identify mutations. Heteroduplex molecules are then separated from homoduplex molecules by ion-pair, reverse-phase liquid chromatography on a column matrix with partial heat denaturation of the DNA strands. The temperature for optimal resolution of heteroduplex and homoduplex DNA was determined by analyzing the melting behavior of a PCR fragment of each exon while the temperature was increased by 1 °C steps from 50 to 55 °C until the fragment was completely melted. The temperature chosen for analysis of each fragment was the point at which 75 % of the DNA was present as an alpha helix or 1 to 2 °C higher [5]. When an aberrant dHPLC pattern was detected, then exon analysis was performed by automatic sequencing on an ABI PRISM 3700 DNA Analyzer using the 3100 BigDye® Terminator v3.1 Cycle Sequencing Kit (Life Technologies, CA, USA).

A 68-year-old woman was diagnosed with metastatic colon carcinoma and treated with irinotecan 180 mg/m^2^, leucovorin 400 mg/m^2^, and 5-FU 400 mg/m^2^ i.v. bolus followed by 2800 mg/m^2^ continuous infusion over 46 h and bevacizumab 5 mg/kg. Toxicity occurred after the first cycle and is described in Table 1. The patient recovered after 23 days, and a second cycle of treatment was started with an empirical dose reduction of both irinotecan (30 %) and 5-FU (75 %). The patient suffered again from toxicity that appeared 7 days from the administration of chemotherapy and recovered after 2 weeks. Treatment was started again with a further dose reduction of 5-FU (85 %), but owing to the poor tolerability, it was eventually discontinued. A second patient was a 57-year-old woman previously diagnosed with a sigmoid carcinoma and given oxaliplatin 85 mg/m^2^, folinic acid 200 mg/m^2^, and 5-FU i.v. bolus 400 mg/m^2^ followed by a 44-h continuous infusion of 400 mg/m^2^ i.v. Toxicity occurred after the second cycle and is described in Table 1. Recovery from toxicity was slow and occurred at day 23. The last case was a 59-year-old woman given adjuvant treatment with capecitabine 1000 mg/m^2^ twice daily for 14 days plus oxaliplatin 130 mg/m^2^ every 3 weeks for a surgically resected B2 colon carcinoma. On day 14 of the first cycle, the patient developed severe gastrointestinal and hematological toxicities (Table 1). Treatment was discontinued and the patient was hospitalized; her toxicity recovered at day 27.

**Table 1:** Type of treatment, genotype, and toxicities graded according to NCI-CTCAE v.4.03 criteria, where 0 is absence of the specific adverse reaction and 4 is the worst grade, in the patients of the present study

Patient	1	2	3
Treatment	Irinotecan 180 mg/m^2^, leucovorin 400 mg/m^2^, and 5-FU 400 mg/m^2^ i.v. bolus followed by 2800 mg/m^2^ continuous infusion over 46 h and bevacizumab 5 mg/kg	Oxaliplatin 85 mg/m^2^, folinic acid 200 mg/m^2^, and 5-FU i.v. bolus 400 mg/m^2^ followed by a 44-h continuous infusion of 400 mg/m^2^ i.v.	Capecitabine 1000 mg/m^2^ twice daily for 14 days plus oxaliplatin 130 mg/m^2^ every 3 weeks
Genotype	c.2509-2510insC	c.85TC	c.680GA
	c.85TC	c.1801GC	c.2194GA
		cc.2194AA	
Toxicity	Grade of severity		
Nausea/vomiting	0	2	4
Diarrhea	4	3	0
Stomatitis	3	4	3
Leukopenia	2	0	4
Neutropenia	2	0	4
Hand-foot syndrome	0	3	1
Anemia	0	0	2
Fever	0	0	4

The procedures described in the present report have been approved by the local ethics committee of University Hospital, Pisa, Italy (protocol n. 3268/2011). All patients were provided with a written detailed description of the experimental procedures of this study and gave their informed consent.

## Results

Several polymorphic variants of *DPYD* have been reported (Table 2). The IVS14+1G>A and c.2846A>T are the most common variants associated with profound DPD deficiency and severe toxicities [6, 7]. However, these mutations were not detected in the present patients. The first subject was heterozygous for the novel c.2509-2510insC and was also carrier of the previously described c.85TC sequence variant. The c.2509-2510insC leads to a leucine-proline substitution in the exon 20 and causes misreading of the coding sequence, altering *DPYD* transcription and enzyme activity. The second patient presented the novel c.1801GC variant, which causes a missense mutation changing a glycine into a serine, as well as the previously described c.85TC, c.1801GC, and cc.2194AA variants. Finally, the third patient carried the novel missense mutation c.680GA, which causes a serine-glycine change, combined with the previously described c.2194GA genotype. Therefore, the insertion c.2509-2510insC as well as the c.1801G>C and c.680G>A are novel mutations not previously reported and are associated with fluoropyrimidine-induced toxicity.

**Table 2:** *DPYD* variants detected in patients with adverse reactions to fluoropyrimidines

Exon/intron	Variant	Reference
2	c.61C>T, c.62G>A, c.74A>G, c.85T>C	[11, 18, 19]
3	c.187A>G	[8]
4	c.257C>T	[19]
5	c.464T>A	[20]
6	c.496A>G, c.601A>C	[19, 21]
7	c.703C>T	[22]
8	c.775A>G, c.812delT	[23, 24]
10	c.1003G>T, c.1039delTG, c.1050G>A, c.1109delTA, c.1108A>G	[8, 11, 19, 21]
11	c.1156G>T	[11]
12	c.1358C>T	[25]
13	c.1590T>C, c.1601G>A, c.1627A>G, c.1679T>G, c.1714C>G	[11, 18, 19, 24, 26]
14	c.1896T>C, c.1897delC	[11, 27]
18	c.2194G>A	[19]
22	c.2846A>T	[13, 18, 21]
23	c.2933A>G	[19]
Intron 6	IVS6-29G>T	[28]
Intron 10	IVS10-15T>C	[21]
Intron 14	IVS14+1G>A	[13, 27]

## Conclusions

The role of *DPYD* deficient variants as a cause of fluoropyrimidine-induced toxicity is still a debated issue [7]. In the present study, we identified three novel mutations within *DPYD*, together with other known sequence variants. The role of c.85T>C is uncertain; in previous studies, c.85T>C has not been correlated with gastrointestinal toxicity [8] and has apparently no functional significance on DPD activity [9]. It is likely that c.85T>C does not cause DPD deficiency by itself [10] but may potentiate the detrimental effect of the genetic variants with which it is combined. The novel c.1801G>C mutation was found in combination with the homozygous c.2194AA; even if the latter is associated with modest 5-FU toxicity [11], the novel mutation could represent a detrimental variant and increase the effect of other single nucleotide polymorphisms. The novel c.680GA was present in the third patient in association with c.2194GA; since c.2194GA has not been implicated in severe toxicities in patients treated with fluoropyrimidines, the new mutation c.680GA could play a critical role in this setting. In each case, a different amino acid is incorporated into the mutated protein as a consequence of the non-synonymous nature of the variants and is expected to have functional consequences. In the first case, bearing the c.2509-2510insC, leucine and proline share some similarities, being both hydrophobic, but the distinctive cyclic structure of proline’s side chain gives this amino acid an exceptional conformational rigidity compared to other amino acids while leucine possesses an aliphatic side chain that is non-linear and prefers to be buried in protein hydrophobic cores. Indeed, their substitution in enzymes can cause profound destabilization [12]. In the second and third cases, the differences in amino acid properties are even more pronounced since serine is a polar amino acid and may participate in hydrogen bonds, while glycine is hydrophobic and is normally buried inside the protein core. The outlined characteristics of the amino acids being substituted in the DPD enzyme provide a justification of the change in DPD activity although a functional enzymatic assay was not performed in these patients because they were lost to follow-up before the enzyme assay was available in our laboratory.

Being the number of deficient variants of DPD quite large, the crucial issue is if it is necessary to investigate all of them [13].

This uncertainty has made a trial-and-error attitude very common in the oncology community, which has followed an empiric approach thus far with respect to prediction and prevention of fluoropyrimidine-induced toxicity in cancer patients. Unfortunately, this approach exposes a significant proportion of patients to the risk of severe and life-threatening toxicities, even though meta-analyses demonstrated the strong association between deficient variants and development of adverse events [14]. Nevertheless the scientific evidence about the usefulness of a targeted pharmacogenetic approach has been clearly recognized [15]. However, the main reason for the limited diffusion of preemptive DPD testing are the costs associated with the analysis [16], owing to the quite large number of deficient *DPYD* variants associated with impaired DPD activity and risk of toxicity.

The issue of application of an optimal preventive, prognostic, and personalized medicine approach to patient care is the object of active discussion and has been addressed by the EPMA in the white paper where a number of recommendations have been made [17]. Therefore, to help implement *DPYD* genotyping in clinical practice, a working group of the Italian Association of Medical Oncology and the Italian Society of Pharmacology (http://www.sifweb.org/docs/sif_aiom_position_paper_raccomand_farmacogen_gen15.pdf, last accessed 22 July 2015) released a statement concerning the screening of major *DPYD* deficient variants, including IVS14+1G>A, c.1679T>G, and c.2846A>T, and recommended it to be performed before treatment with fluoropyrimidines, whenever the proposed treatment is planned in one or more of these conditions: (a) patient at risk (i.e., comorbidity, performance status, disease stage), (b) limited therapeutic advantage in terms of survival and/or response, (c) high ratio of risk/benefits or (d) in case of *G* ≥ 3 gastrointestinal or *G* = 4 hematological toxicities (NCI-CTCAE v4.0) during fluoropyrimidine treatment, and (e) in every case of unexpected adverse reaction.

In conclusion, the novel polymorphisms detected in these subjects increase our knowledge about the causes of unexpected life-threatening toxicities in patients treated with fluoropyrimidines. The effort of future studies will be the appropriate identification of at risk mutations in order to avoid inappropriate drug dosing in poor-metabolizer patients candidate to adjuvant fluoropyrimidine treatment and implement their preemptive screening in current clinical practice.

## Expert recommendations

The present manuscript describes three novel variants of *DPYD* associated with unexpected severe toxicities in patients treated with fluoropyrimidines. It is suggested that, as a strategic component of the preventive, prognostic, and personalized approach to improve the efficacy vs. toxicity ratio, a preemptive screening of deficient DPD variants should be performed in patients as a part of standard healthcare. However, due to the considerable number of *DPYD* mutations and to balance the costs vs. benefits, the analysis of the most frequent variants associated with DPD deficiency, i.e., IVS14+1G>A, c.1679T>G, and c.2846A>T, should be performed before treatment, while the detection of additional rare mutations is recommended in patients suffering from unexpected severe toxicities early after the beginning of treatment, until more affordable technological platforms will allow a cost-effective analysis of all deficient variants associated with the poor-metabolizer status.

## Abbreviations

5′-DFCR: 5′-deoxy-5-fluorocytidine
5′-DFUR: 5′-deoxy-5-fluorouridine
5-FU: 5-fluorouracil
dHPLC: denaturing HPLC
DPD: dihydropyrimidine dehydrogenase enzyme
*DPYD*: dihydropyrimidine dehydrogenase gene
PCR: polymerase chain reaction
UFT: ftorafur/uracil

## References

[1] Walko CM, Lindley C (2005). Capecitabine: a review. Clin Ther.

[2] Tanaka F, Fukuse T, Wada H, Fukushima M (2000). The history, mechanism and clinical use of oral 5-fluorouracil derivative chemotherapeutic agents. Curr Pharm Biotechnol.

[3] Johnson MR, Hageboutros A, Wang K, High L, Smith JB, Diasio RB (1999). Life-threatening toxicity in a dihydropyrimidine dehydrogenase-deficient patient after treatment with topical 5-fluorouracil. Clin Cancer Res.

[4] Amstutz U, Froehlich TK, Largiadèr CR (2011). Dihydropyrimidine dehydrogenase gene as a major predictor of severe 5-fluorouracil toxicity. Pharmacogenomics.

[5] Keller G, Hartmann A, Mueller J, Höfler H (2001). Denaturing high pressure liquid chromatography (DHPLC) for the analysis of somatic p53 mutations. Lab Invest.

[6] Saif MW, Ezzeldin H, Vance K, Sellers S, Diasio RB (2007). DPYD*2A mutation: the most common mutation associated with DPD deficiency. Cancer Chemother Pharmacol.

[7] van Kuilenburg AB, De Abreu RA, van Gennip AH (2003). Pharmacogenetic and clinical aspects of dihydropyrimidine dehydrogenase deficiency. Ann Clin Biochem.

[8] Kleibl Z, Fidlerova J, Kleiblova P, Kormunda S, Bilek M, Bouskova K (2009). Influence of dihydropyrimidine dehydrogenase gene (DPYD) coding sequence variants on the development of fluoropyrimidine-related toxicity in patients with high-grade toxicity and patients with excellent tolerance of fluoropyrimidine-based chemotherapy. Neoplasma.

[9] Johnson MR, Wang K, Diasio RB (2002). Profound dihydropyrimidine dehydrogenase deficiency resulting from a novel compound heterozygote genotype. Clin Cancer Res.

[10] Collie-Duguid ESR, Etienne MC, Milano G, McLeod HL (2000). Known variant DPYD alleles do not explain DPD deficiency in cancer patients. Pharmacogenetics.

[11] He YF, Wei W, Zhang X, Li YH, Li S, Wang FH (2008). Analysis of the DPYD gene implicated in 5-fluorouracil catabolism in Chinese cancer patients. J Clin Pharm Ther.

[12] Newbold RJ, Deery EC, Walker CE, Wilkie SE, Srinivasan N, Hunt DM (2001). The destabilization of human GCAP1 by a proline to leucine mutation might cause cone-rod dystrophy. Hum Mol Genet.

[13] Yen JL, McLeod HL (2007). Should DPD analysis be required prior to prescribing fluoropyrimidines?. Eur J Cancer.

[14] Terrazzino S, Cargnin S, Del Re M, Danesi R, Canonico PL, Genazzani AA (2013). DPYD IVS14+1G>A and 2846A>T genotyping for the prediction of severe fluoropyrimidine-related toxicity: a meta-analysis. Pharmacogenomics.

[15] Innocenti F. DPYD variants to predict 5-FU toxicity: the ultimate proof. J Natl Cancer Inst. 2014;106. doi:10.1093/jnci/dju351. 10.1093/jnci/dju351 25381394

[16] Goldstein DA, Shaib WL, Flowers CR (2015). Costs and effectiveness of genomic testing in the management of colorectal cancer. Oncology (Williston Park).

[17] Golubnitschaja O, Costigliola V, EPMA (2012). General report & recommendations in predictive, preventive and personalised medicine 2012: white paper of the European Association for Predictive, Preventive and Personalised Medicine. EPMA J.

[18] Zhang H, Li YM, Zhang H, Jin X (2007). DPYD*5 gene mutation contributes to the reduced DPYD enzyme activity and chemotherapeutic toxicity of 5-FU: results from genotyping study on 75 gastric carcinoma and colon carcinoma patients. Med Oncol.

[19] van Kuilenburg AB, Haasjes J, Meinsma R, Waterham HR, Vreken P, van Gennip AH (2000). Dihydropyrimidine dehydrogenase (DPD) deficiency: novel mutations in the DPD gene. Adv Exp Med Biol.

[20] Morel A, Boisdron-Celle M, Fey L, Lainé-Cessac P, Gamelin E (2007). Identification of a novel mutation in the dihydropyrimidine dehydrogenase gene in a patient with a lethal outcome following 5-fluorouracil administration and the determination of its frequency in a population of 500 patients with colorectal carcinoma. Clin Biochem.

[21] Gross E, Busse B, Riemenschneider M, Neubauer S, Seck K, Klein HG (2008). Strong association of a common dihydropyrimidine dehydrogenase gene polymorphism with fluoropyrimidine-related toxicity in cancer patients. PLoS One.

[22] Shahrokni A, Rajebi MR, Harold L, Saif MW (2009). Cardiotoxicity of 5-fluorouracil and capecitabine in a pancreatic cancer patient with a novel mutation in the dihydropyrimidine dehydrogenase gene. JOP.

[23] Vreken P, van Kuilenburg ABP, Meinsma R, De Abreu RA, VanGennip AH (1997). Dihydropyrimidine dehydrogenase (DPD) deficiency: identification and expression of missense mutations C29R, R886H and R235W. Hum Genet.

[24] Morel A, Boisdron-Celle M, Fey L, Soulie P, Craipeau MC, Traore S (2006). Clinical relevance of different dihydropyrimidine dehydrogenase gene single nucleotide polymorphisms on 5‐fluorouracil tolerance. Mol Cancer Ther.

[25] Gross E, Ullrich T, Seck K, Mueller V, de Wit M, von Schilling C (2003). Detailed analysis of five mutations in dihydropyrimidine dehydrogenase detected in cancer patients with 5‐fluorouracil‐related side effects. Hum Mutat.

[26] van Kuilenburg AB, Haasjes J, Richel DJ, Zoetekouw L, Van Lenthe H, De Abreu RA (2000). Clinical implications of dihydropyrimidine dehydrogenase (DPD) deficiency in patients with severe 5-fluorouracil-associated toxicity: identification of new mutations in the DPD gene. Clin Cancer Res.

[27] Mercier C, Ciccolini J (2007). Severe or lethal toxicities upon capecitabine intake: is DPYD genetic polymorphism the ideal culprit?. Trends Pharmacol Sci.

[28] Ben Fredj R, Gross E, Chouchen L, B'Chir F, Ben Ahmed S, Neubauer S (2007). Mutational spectrum of dihydropyrimidine dehydrogenase gene (DPYD) in the Tunisian population. C R Biol.

